# COGNITIVE AND FUNCTIONAL IMPACTS OF CHILD LABOR IN INDIA: THE MEDIATING ROLE OF EDUCATION

**DOI:** 10.1093/geroni/igad104.3076

**Published:** 2023-12-21

**Authors:** Seolah Lee, Kriti Vikram, Hyo Jung Lee

**Affiliations:** Yonsei University, Seoul, Republic of Korea; National University of Singapore, Singapore, Singapore; Yonsei University, Seoul, Republic of Korea

## Abstract

This study aims to examine how entry into the labor market in childhood is associated with deficits in late-life cognitive and functional health. Using the Cumulative Advantage/Disadvantage Theory, it further explores how educational attainment, an indicator of socio-economic status, mediates the associations between child labor and cognitive and functional health among older adults in India. Lastly, it investigates if the results vary by gender. We use the nationally representative World Health Organization Study on global AGEing and Health (SAGE), and select respondents aged 50 years or older (n = 6,215 for Mini-Mental State Examination (MMSE); n=6,408 for the Functional Limitation (FL)). Mediation models were performed for each dependent variable using the sgmediation2 program, bootstrapped 5,000 resamples, and percentile bootstrap confidence interval. The indirect and total effects of child labor on MMSE were significant, while the direct effect of child labor was not. For FL, the indirect effect was significant, but the direct and total effects were not. Gender difference was found in terms of the direct effect, child work was directly associated with a lower MMSE score in older women (p < .01), whereas it was not significant for older men. Findings suggest that early entry into the labor market (as a child) has a negative impact on older adults’ MMSE and FL through deprivation of educational opportunities. Providing opportunities for completing certain level of education can mitigate the negative impact of child labor on health in old age.

